# Evaluation of eGFP expression in the ChAT-eGFP transgenic mouse brain

**DOI:** 10.1186/s12868-023-00773-9

**Published:** 2023-01-17

**Authors:** Rashmi Gamage, Laszlo Zaborszky, Gerald Münch, Erika Gyengesi

**Affiliations:** 1 Pharmacology Unit, Group of Pharmacology, School of Medicine, Western Sydney University, Penrith, NSW 2751 Australia; 2 Center for Molecular and Behavioral Neuroscience, Rutgers The State University of New Jersey, Newark, NJ 07102 USA

**Keywords:** Transgenic mice, ChAT eGFP, Immunohistochemistry, Basal forebrain cholinergic neurons, Antibody staining, In situ hybridization, Allen mouse brain atlas, Choline acetyltransferase

## Abstract

**Background:**

A historically definitive marker for cholinergic neurons is choline acetyltransferase (ChAT), a synthesizing enzyme for acetylcholine, (ACh), which can be found in high concentrations in cholinergic neurons, both in the central and peripheral nervous systems. ChAT, is produced in the body of the neuron, transported to the nerve terminal (where its concentration is highest), and catalyzes the transfer of an acetyl group from the coenzyme acetyl-CoA to choline, yielding ACh. The creation of bacterial artificial chromosome (BAC) transgenic mice that express promoter-specific fluorescent reporter proteins (green fluorescent protein—[GFP]) provided an enormous advantage for neuroscience. Both in vivo and in vitro experimental methods benefited from the transgenic visualization of cholinergic neurons. Mice were created by adding a BAC clone into the ChAT locus, in which enhanced GFP (eGFP) is inserted into exon 3 at the ChAT initiation codon, robustly and supposedly selectively expressing eGFP in all cholinergic neurons and fibers in the central and peripheral nervous systems as well as in non-neuronal cells.

**Methods:**

This project systematically compared the exact distribution of the ChAT-eGFP expressing neurons in the brain with the expression of ChAT by immunohistochemistry using mapping and also made comparisons with in situ hybridization (ISH).

**Results:**

We qualitatively described the distribution of ChAT-eGFP neurons in the mouse brain by comparing it with the distribution of immunoreactive neurons and ISH data, paying special attention to areas where the expression did not overlap, such as the cortex, striatum, thalamus and hypothalamus. We found a complete overlap between the transgenic expression of eGFP and the immunohistochemical staining in the areas of the cholinergic basal forebrain. However, in the cortex and hippocampus, we found small neurons that were only labeled with the antibody and not expressed eGFP or vice versa. Most importantly, we found no transgenic expression of eGFP in the lateral dorsal, ventral and dorsomedial tegmental nuclei cholinergic cells.

**Conclusion:**

While the majority of the forebrain ChAT expression was aligned in the transgenic animals with immunohistochemistry, other areas of interest, such as the brainstem should be considered before choosing this particular transgenic mouse line.

## Background

Since the generations of the first transgenic mouse line in 1989 by Gordon et al., the technique of introducing a foreign gene into a mouse oocyte using pronuclear injection has become wide spread, resulting in the generation of hundreds of different mouse lines [1]. Thus, with meticulous technique, any cloned-gene sequence could be integrated into the DNA of rodents, and, once injected, these genes inserted into the host DNA at random sites, being dispersed throughout the genome. Due to the development in molecular biological techniques and genetics, genes can be inserted, deleted, multiplied, and conditionally expressed. Combined with another revolutionary development in neuroscience the expression of green fluorescent protein (GFP) in the mammalian brain [2] the scientific community gained ready access to visualizations of certain cell types in the vertebrae nervous system without using immunohistochemistry. GFP is a naturally fluorescent gene product of the bioluminescent jellyfish *Aequorea victoria*. Since the native GFP protein is not particularly bright (excited by UV light at 395 nm), scientists were more interested in the genetically engineered versions, such as the enhanced GFP (eGFP), with red-shifted excitation maxima (induced using lasers emitting at 488 nm) and increased quantum efficiencies (up to 35-fold) [3]. Thus, the eGFP version was much more useful in fluorescence and confocal microscopy. These techniques without a doubt contributed to significant discoveries about the structure and function of the central nervous system. Today there are 644 mouse strains available in stock at Jacksons Laboratories that contain a fluorescent protein insert [4].

The basal forebrain (BF) cholinergic system has been the center of attention over the past 30 years—first because of its role in the regulation of the sleep–wake cycle and cortical activation and also because of its possible connection with Alzheimer’s disease and other dementias [5–9]. Cholinergic neurons were traditionally visualized by immunohistochemistry; antibodies were used against choline acetyltransferase (ChAT), which is essential for acetylcholine (ACh) synthesis [10, 11]. However, in the past decades, there has been an expansion in the creation of transgenic animals—a powerful new tool for visualizing certain types of neurons or proteins. The cloning of the ChAT^BAC^- eGFP mouse not only allowed scientists to immediately visualize the cholinergic cell population under epifluorescent light but also made targeted electrophysiological recordings and embryological, developmental examinations possible [12–14]. The ChAT^BAC^- eGFP mouse is widely used now in investigations of the central nervous system, spinal-cord motor, and interneurons in adults and embryos, retinal cells, and auditory tubes [15–18]. The bacterial artificial chromosome (BAC) transgenic approach that utilized the ChAT locus to direct expression of eGFP has been described [19, 20]. Nevertheless, the marker expression in peripheral cholinergic neurons was not reported in these mice, which prevented determination of the effectiveness of the BAC strategy in the periphery.

To date, a comprehensive report about the distribution of ChAT promoter driven eGFP expression in the entire mouse brain has not been described in detail. To confirm the expression of ChAT-eGFP in the entire mouse brain, here we report the precise qualitative distribution of neurons that express the inserted eGFP and compare that distribution to the traditional immunohistochemical method of labeling ChAT, as well as with the in situ hybridization (ISH) of the ChAT mRNA expression published by the Allen Mouse Brain Atlas [21].

## Results

To investigate the distribution of ChAT-eGFP-positive cells in the adult-mouse brain, we individually mapped and analyzed series of sections from the entire mouse brain for neurons that contain ChAT. These were visualized either by transgenic eGFP protein expression, immunohistochemistry, or ISH, published by the Allen Institute for Brain Science [21]. We manually mapped 44 sections, starting from + 2.40 mm from the bregma to − 6.4 mm, using MBF Neurolucida software, covering the entire brain in coronal sections and compared the localization and expression patterns of the cholinergic neurons that expressed the eGFP tag, antibody, or ChAT RNA.

### Forebrain regions that contain ChAT-positive Neurons

#### Telencephalon

**Fig. 1:**
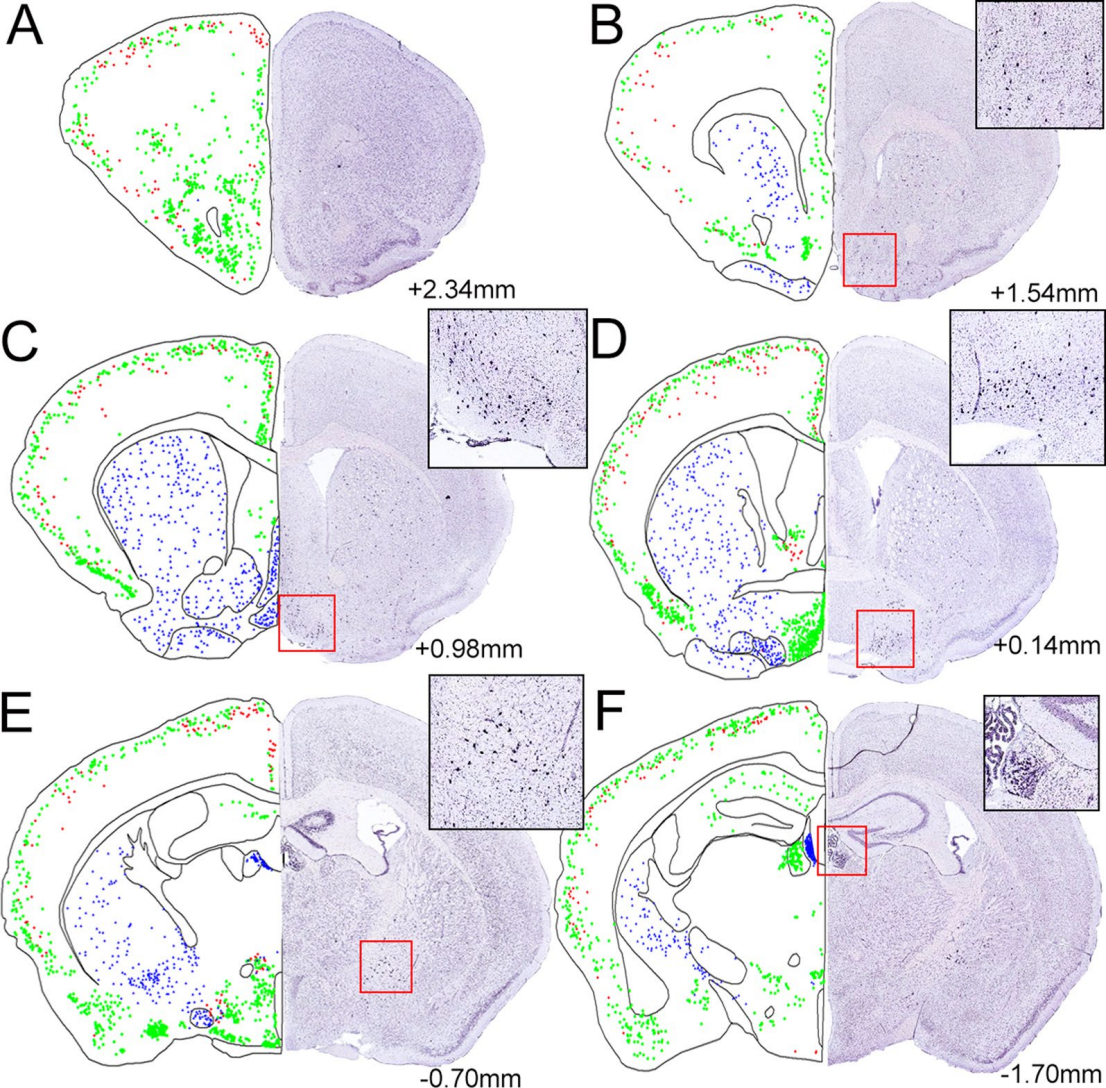
Shown are representative examples of mapped cholinergic neurons, between the areas of + 2.34 mm to − 1.7 mm from the bregma (A-F), labeled by either ChAT-eGFP expression (green), ChAT antibody labeled (red), or neurons expressing both eGFP and antibody staining (blue) on the left side of the panels. The right side of each panel and inserts represent ChAT ISH images modified from the Allan Institute for Brain Science. Allen reference atlas—mouse brain [gene search], https://mouse.brain-map.org/gene/show/12432

A scattered pattern of both ChAT-eGFP- and Gt-a-ChAT-labeled neurons was found throughout the prefrontal cortical areas. ChAT-eGFP neurons were concentrated in the anterior olfactory area, medial, and posterior parts as well as in the endopiriform claustrum (En). Both ChAT-eGFP and antibody labeled neurons were sparsely dispersed in the cortical areas, including the orbital (O), the prelimbic (PrL), infralimbic (Il), cingulate (Cg), motor (M), somatosensory (S), insular (I), and dense-cell-layer piriform (Pir) cortices. In the cortical areas, the two markers showed very low overlap at any levels (Figs. 1A, 3A–C).The dorsal tenia tecta (DTT) contained ChAT-eGFP neurons, but no antibody labelled neurons (Fig. 1B). Consistent with these observations, ISH data represented similar scattered ChAT mRNA expression in the isocortex and olfactory areas, as shown in comparative right-hand-side image panels in Fig. 1.

The medial septum (MS), nucleus of the horizontal, ventral and lateral limb of the diagonal band (HDB/VDB/LDB), ventral pallidum (VP), caudate putamen (CPu), globus pallidus (GP), and the extension of the amygdala (EA) only contained double-labeled neurons in their entire expansion (Figs. 1C–D, 3 D–I). We also found double-labeled neurons in the tuberculum (Tu) (Fig. 1C–D). Throughout the accumbens nucleus (ACB) shell ChAT-eGFP/Gt-a-ChAT-positive double-labeled neurons were found (Fig. 1C–D). Consistent with these observations, ISH data represented similar high ChAT mRNA expression in the entire BF expansion, Tu, ACB shell, and striatum as shown in comparative right-hand-side image panels in Fig. 1.

**Fig. 2:**
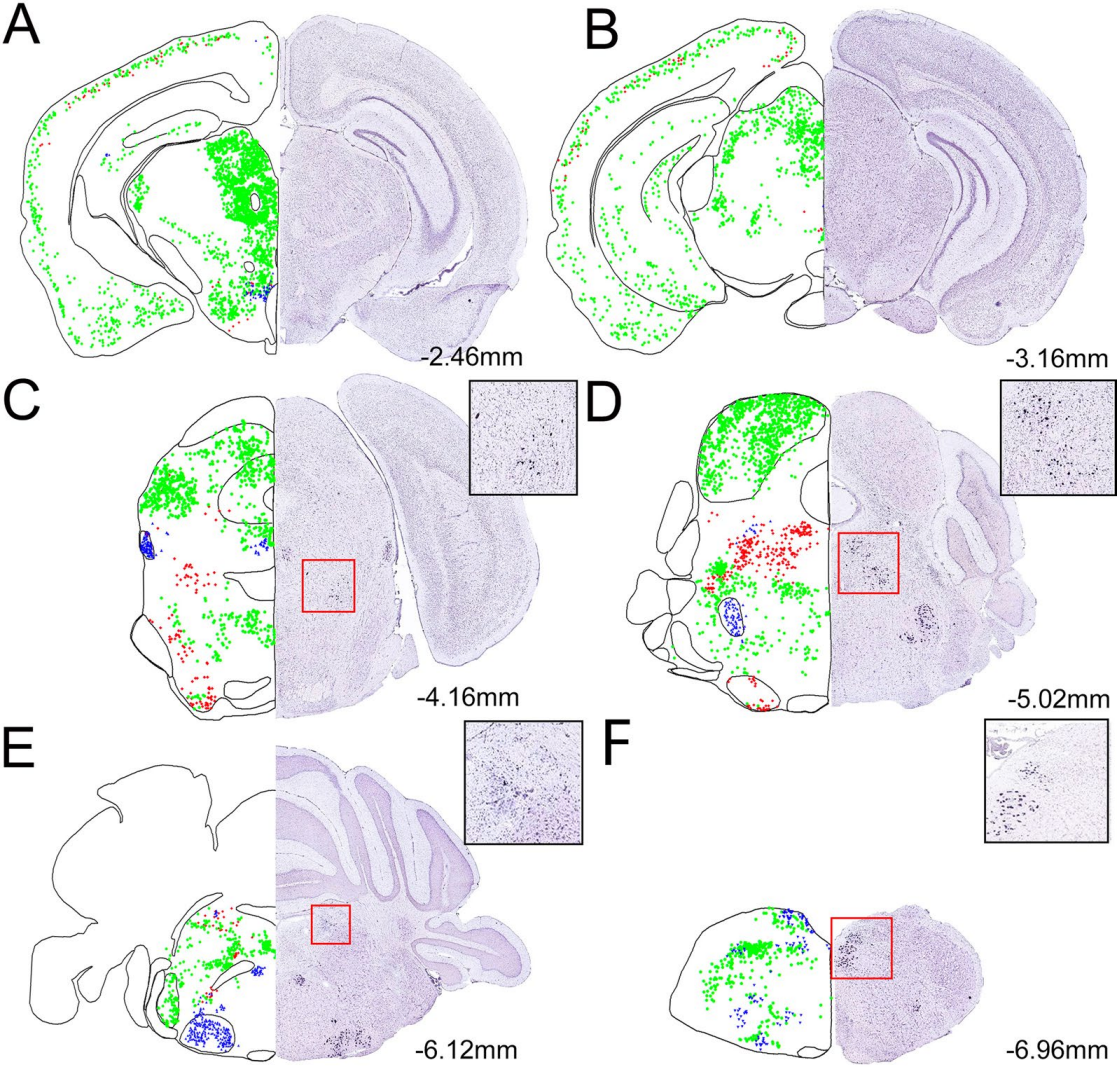
Shown are representative examples of mapped cholinergic neurons, between the areas of − 2.46 mm to − 7.0 mm from the bregma (A-F), labeled by either ChAT-eGFP expression (green), ChAT antibody labeled (red), or neurons expressing both eGFP and antibody staining (blue) on the left side of the panels. The right side of each panel and inserts represent ChAT ISH images modified from the Allan Institute of Brain Science. Allen reference atlas—mouse brain [gene search], https://mouse.brain-map.org/gene/show/12432

Occasionally, ChAT-eGFP neurons were found in the hippocampus cornu ammonis (CA1-3) and dentate gyrus (DG) regions, but appearances of the positively antibody- labeled cells were extremely sporadic (Figs. 1E–F, 2A–B, 4A–I). ISH ChAT mRNA expression levels were consistent with ChAT-eGFP expression patterns in the hippocampus, as shown in comparative right-hand-side image panels in Figs. 1E–F and 2A–B.

**Fig. 3:**
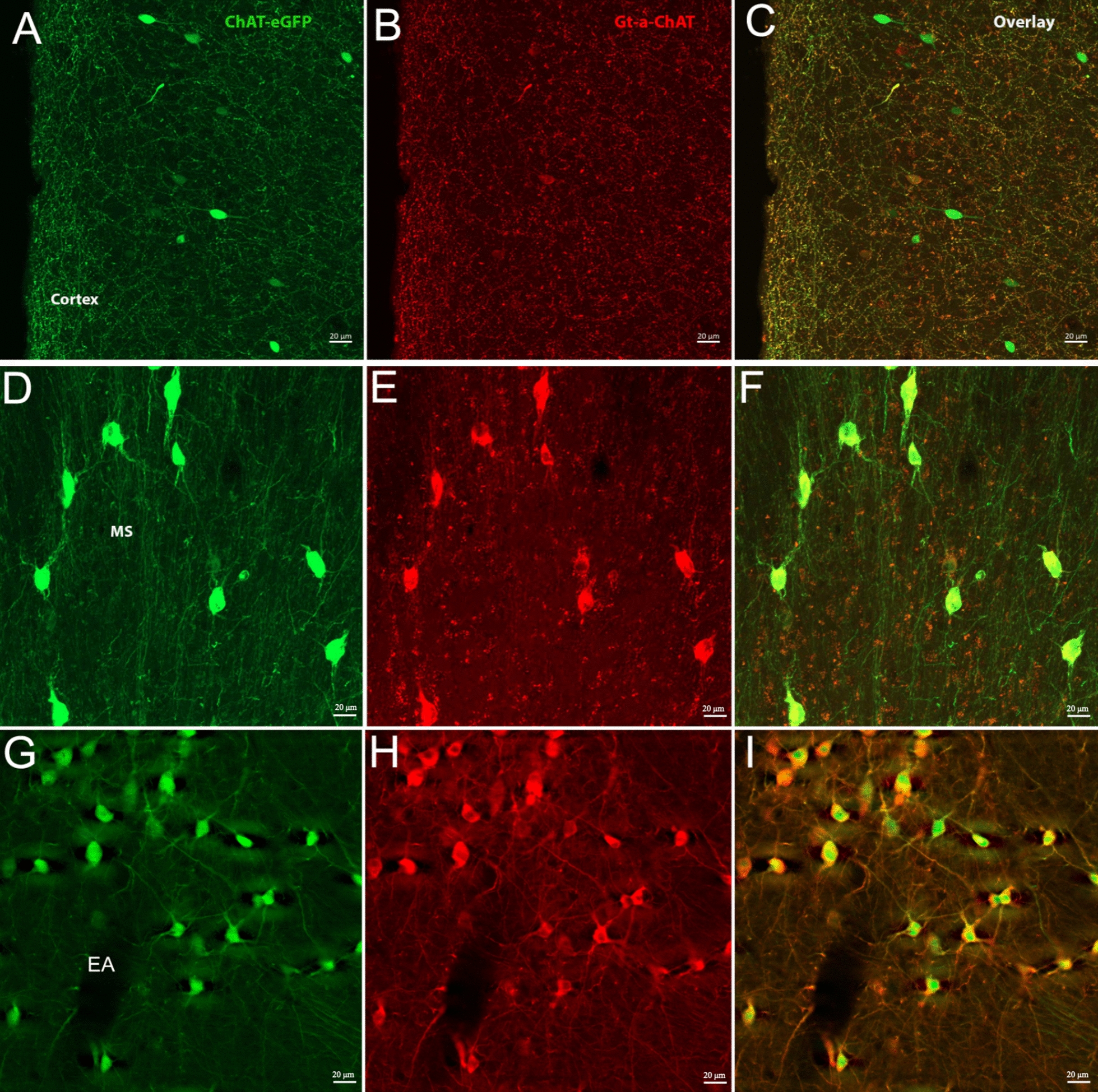
Shown are representative images of cholinergic neurons in the cortex (**A**–**C**) and the BF MS (**D**–**F**) and extended amygdala (**G**–**I**). The scale bar represents 20 µm

**Fig. 4:**
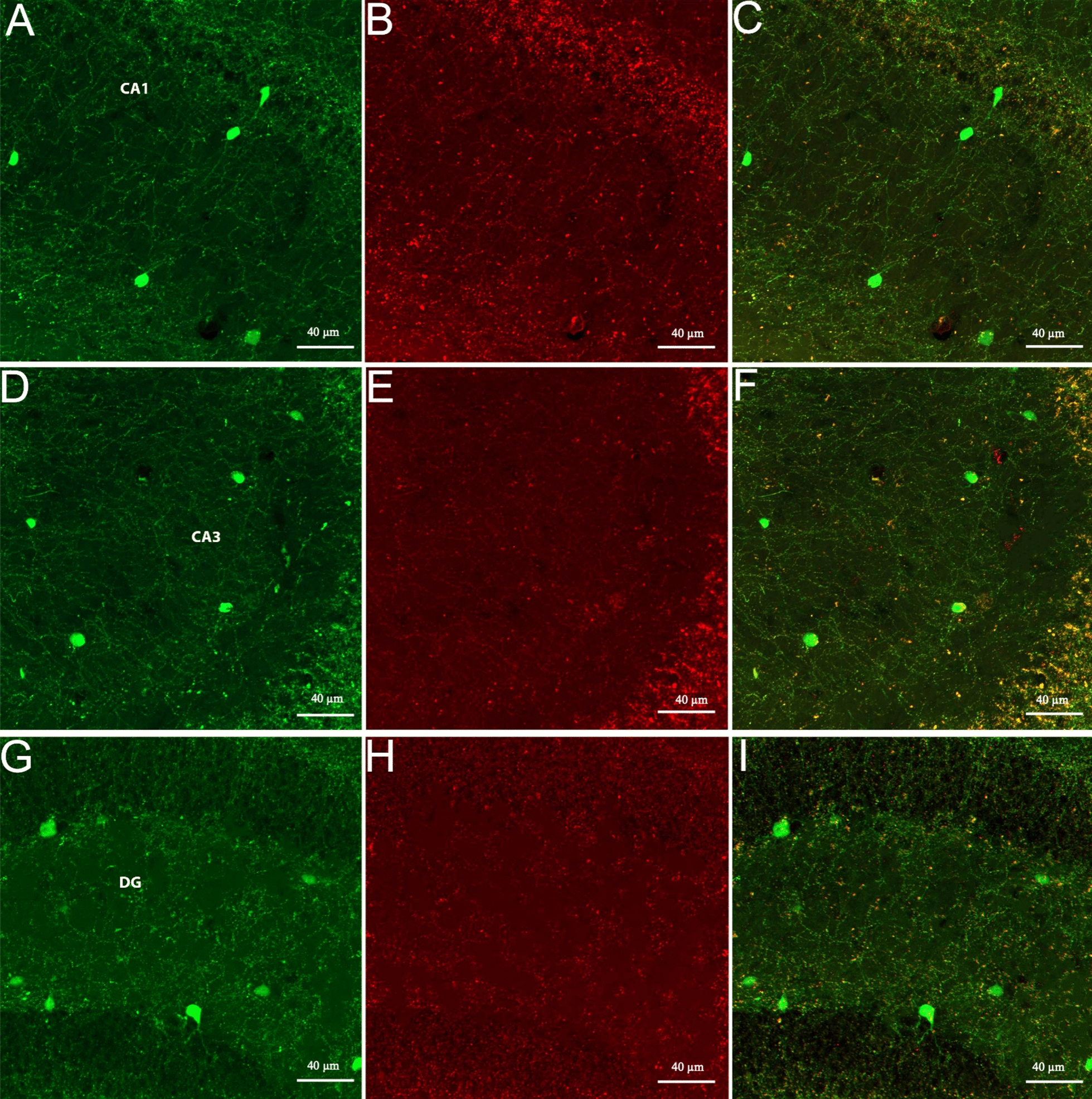
Shown are representative images of cholinergic neurons in the hippocampus, including the CA1 (**A**–**C**), CA3 (**D**–**F**) and DG (**G**–**I**). The scale bar represents 40 µm

Among the amygdaloid regions, the medial amygdala including the anterior, posterodorsal, posteroventral, and the basomedial amygdala nucleus were labeled with ChAT-eGFP but not with the antibody (Figs. 1E–F, 2A). ISH mRNA expression was high in the amygdaloid regions and was similar to the ChAT-eGFP expression patterns as shown in the comparative right-hand-side image panels in Figs. 1 and 2.

### Diencephalon

Significant numbers of ChAT-eGFP positive neurons were located at the LOT2, and they were not detected by using the antibody. In addition to the BF areas, the medial habenula (MHb) also contained double-labeled neurons, but, interestingly, the lateral habenula (LHb) only contained neurons labeled with ChAT-eGFP in the transgenic mice (Fig.1F). Consistent with ChAT-eGFP expression, ISH data show ChAT mRNA expression in both MHb and LHb.

In the thalamus, ChAT-eGFP neurons can be found in the parafascicular (Pf), posterior (Po), pretectal (PoT), and posteromedial areas (PoM) (Fig. 2A). In the zone incerta (ZI), which is an extension of the reticular nucleus of the thalamus, we found a high number of ChAT-eGFP-positive neurons throughout the structure but no overlap with the antibody reaction (Fig. 1E). ISH data was consistent with these observations, indicating ChAT mRNA expression in the thalamic expansion, including the ZI (Figs. 1E–F, 2A–B).

**Fig. 5:**
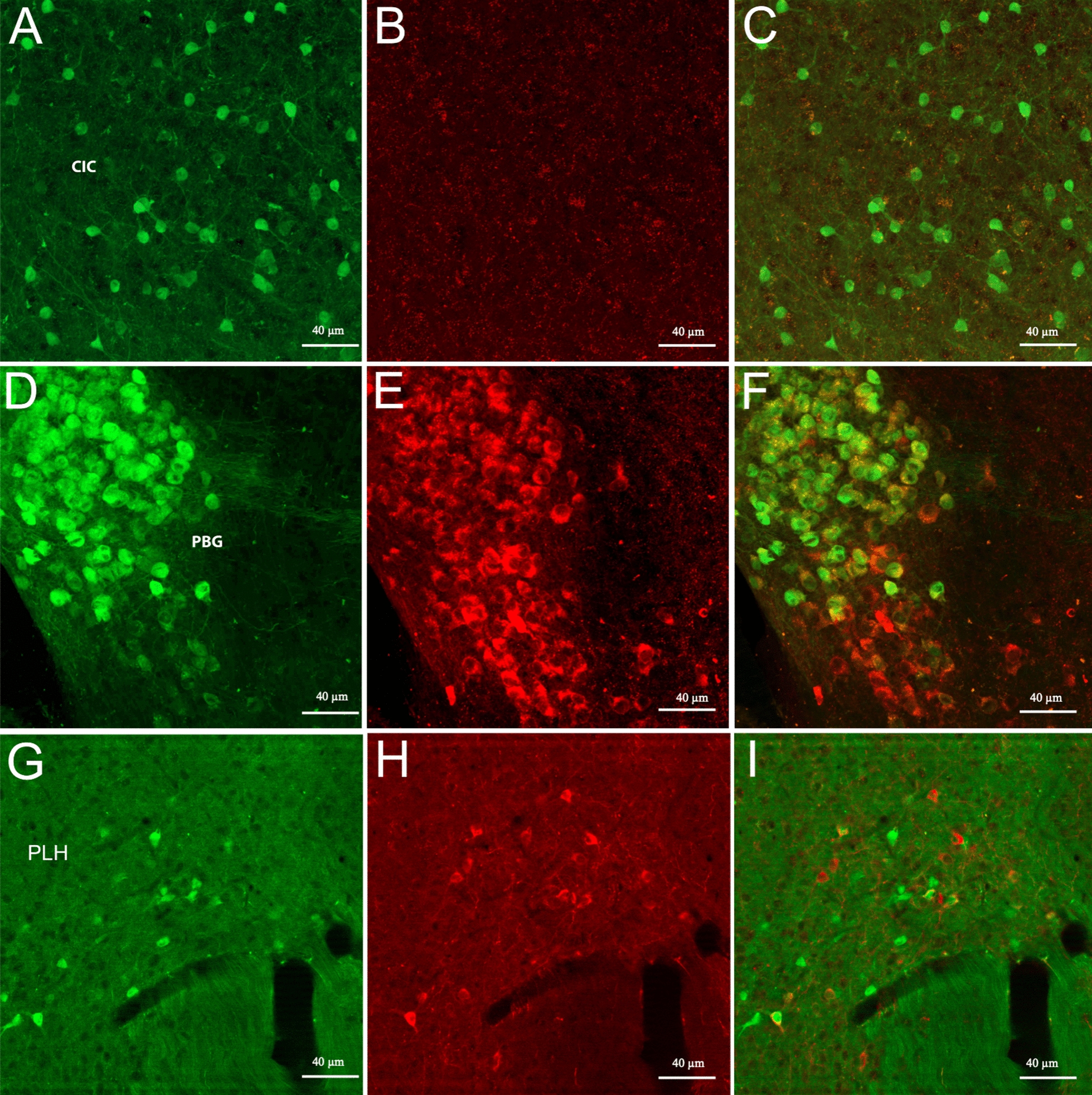
Shown are representative images of cholinergic neurons in the inferior colliculus (CIC) (**A**–**C**), parabigeminal nucleus (PBG) (**D**–**F**), and the peduncular part of the lateral hypothalamus (PLH) (**G**–**I**). The scale bar represents 40 µm

The preoptic areas and the hypothalamus were heavily populated with small, ChAT-eGFP neurons that were not detected by the antibody. In the hypothalamus, the lateral anterior hypothalamus (LAH), ventrolateral hypothalamus (VLH), anterior hypothalamic nucleus (AHC), lateral hypothalamus (LH) and posterior lateral hypothalamus (PLH) areas were strongly ChAT-eGFP labeled (Figs. 1E–F, 2A–B, 5G–I). We also observed ChAT mRNA expression in these hypothalamic regions. In the arcuate nucleus (Arc) we found neurons labeled by only the antibody but not the eGFP (Fig. 1 F). ISH data were consistent with the antibody labeling and we observed ChAT mRNA expression in the Arc.

### Midbrain and hindbrain regions that contains ChAT-positive neurons

**Fig. 6:**
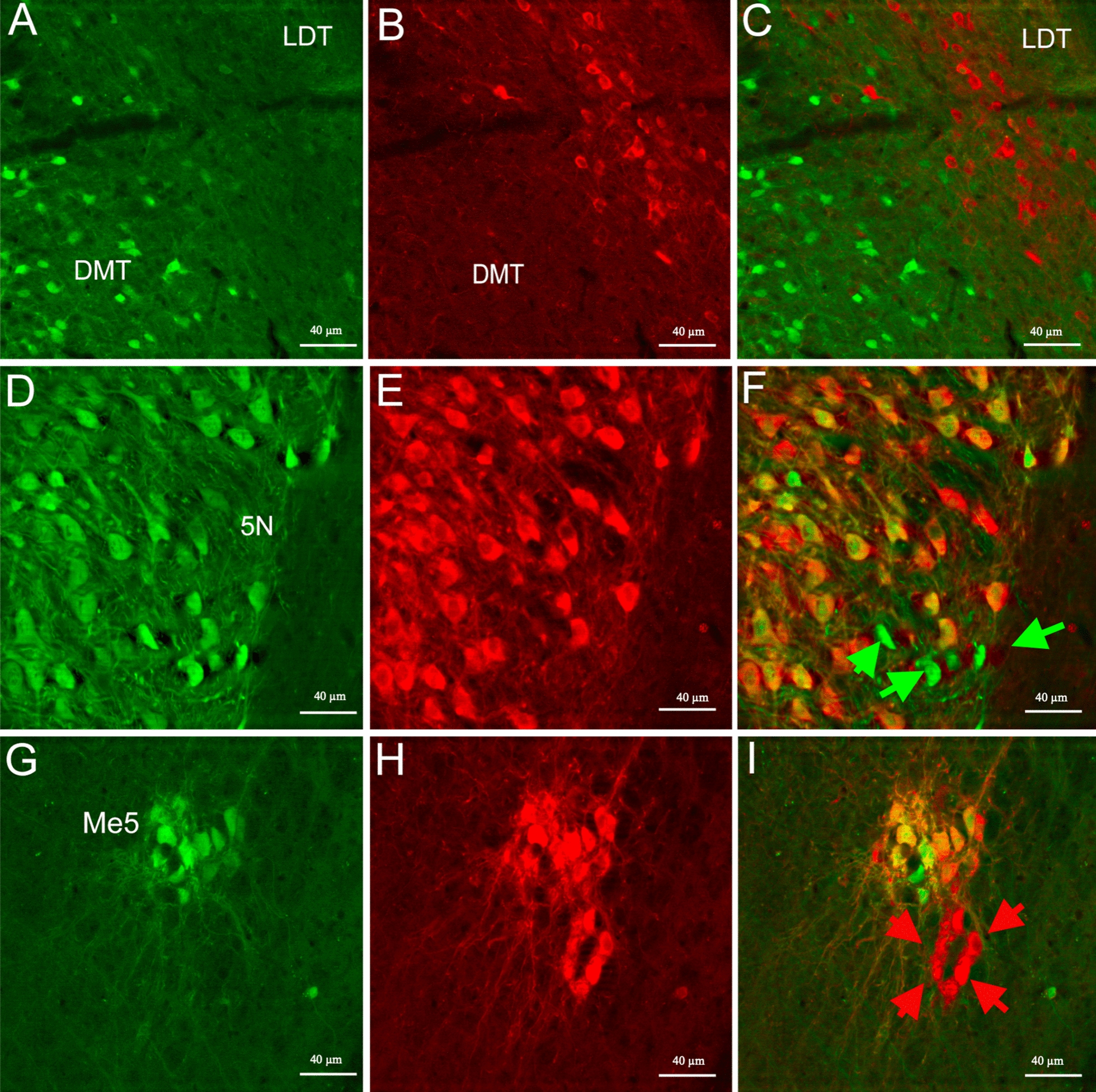
Shown are representative images of cholinergic neurons in the DMT and LDT (**A**–**C**), motor trigeminal nucleus (5 N) (**D**–**F**), and), Me5 (**G**–**I**). Green arrows are areas that express ChAT-eGFP only; red arrows are areas immunostained with Gt-a-ChAT only. The scale bar represents 40 µm

Double labeled and ISH ChAT mRNA-expressing neurons were located in the pedunculotegmental areas (PTg), parabigeminal nucleus (PBG) (Figs. 2C, 5D–F), the cranial nerve nuclei, including oculomotor nucleus (3 N), motor trigeminal nucleus (5 N) (Figs. 2D–E, 6D–F), facial nucleus (7 N), abducens nucleus (6 N), dorsal motor nucleus of the vagus (10 N), and hypoglossal nucleus (12 N) (Fig. 2D–E). The nucleus ambiguous (NA) also contained both eGFP and immunoreactive ChAT antibody labeled cells (Fig. 2E–F).

A population of ChAT-eGFP and ISH ChAT mRNA-expressing neurons was found in the inferior colliculus (CIC) but not labeled by the antibody (Figs. 2D, 5A–C).

We found areas that revealed cholinergic neurons by using immunohistochemistry and ISH but only in the lateral dorsal tegmental (LDT) and ventral tegmental (VT) nucleus (Fig. 2D). A small population of ChAT-eGFP-expressing neurons was found in the dorsomedial tegmental (DMT) nucleus (Figs. 2D, 6A–C).

Also found were both ChAT-immunostained and ChAT-eGFP-expressing neuronal populations in the mesencephalic trigeminal nucleus (Me5) consistent with ISH ChAT mRNA-expression data (Figs. 2D, 6G–I).

### Summary of anatomical areas with ChAT-eGFP and antibody expression

In summary, there was almost perfect overlap in the BF and striatal areas or, more precisely, in the MS, VDB/HDB/LDB, VP, EA, and in the CPu, GP, and interstitial nucleus of the posterior limb of the anterior commissure (IPAC) (Figs. 1C–D, 3D–F). Only a few regions, such as the PLH, PBG, 5 N, and Me5, had cells with antibody labeling or eGFP expression only as well as overlap of both. Moreover, both antibody labeling and ChAT-eGFP expression in transgenic mice were consistent with ISH ChAT mRNA-expression data.

**Fig. 7:**
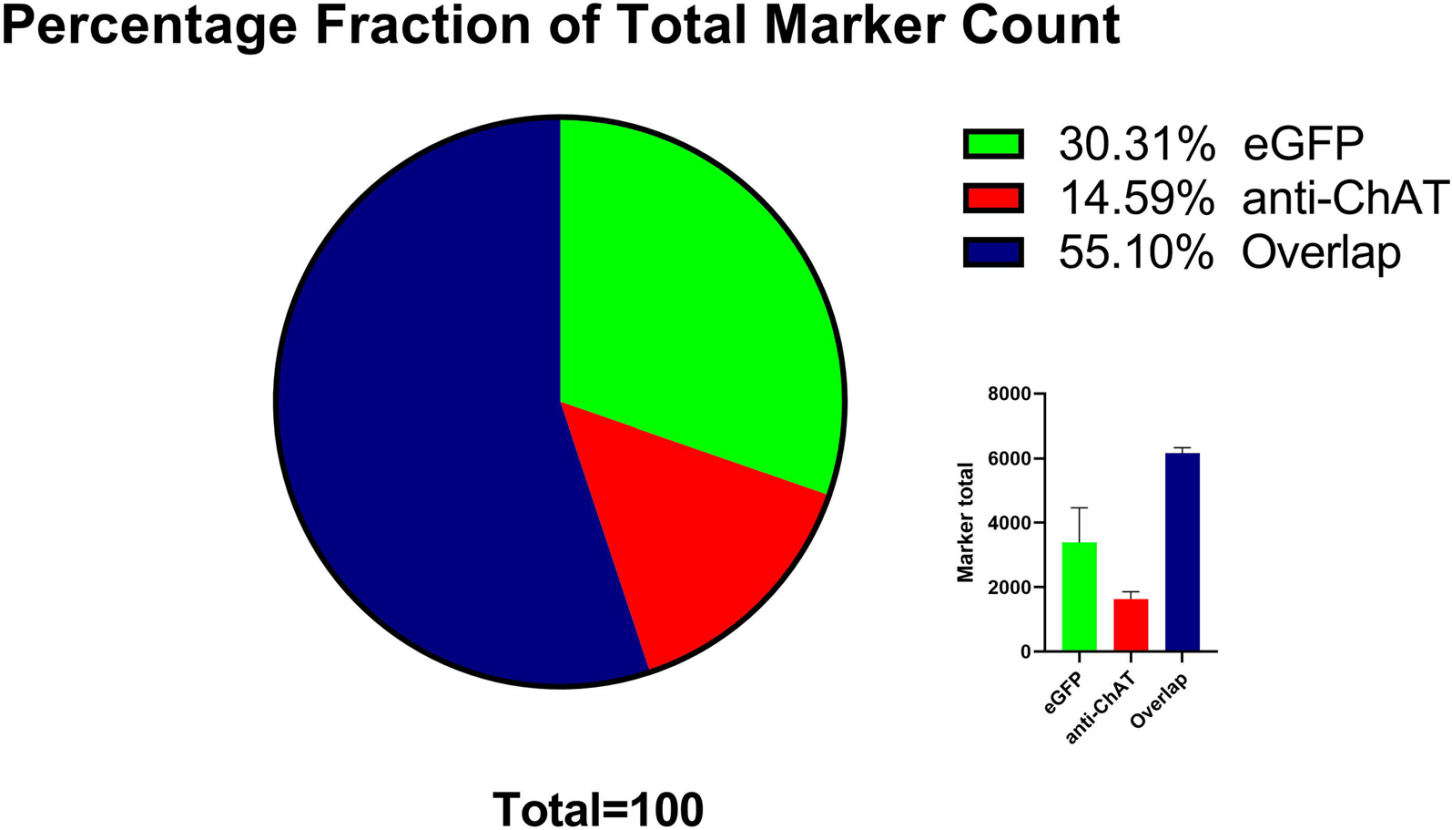
Shown is a representative pie chart of total markers counted, as a percentage of the whole. Marker percentage of eGFP expression (green), anti-ChAT (red), and overlap of the two markers (blue) are represented after mapping the whole mouse brain, sectioned at 40 µm into three series

To summarize the results, we found prominent ChAT-eGFP expression, without corresponding ChAT-antibody binding, in the preoptic areas, including DTT, lateral septum, and LHb. Also found were occasional but consistent numbers of neurons in the hippocampus (CA1-CA3, DG) (Fig. 4A–I), thalamus, periaqueductal gray, and vestibular nucleus. The CIC of the midbrain (Figs. 2D,  5A–C) and the DMT of the midbrain nuclei (Figs. 2D, 6A–C) also showed significant ChAT-eGFP expressing neurons. High-intensity antibody labeling of ChAT neurons in the LDT was observed as well as a secluded subpopulation of cells in Me5 that only expressed ChAT immunoreactive cells (Fig. 6G–I). After mapping the two transgenic mouse brains manually, we found that double-labeled (blue marker) cells that contained both eGFP^+^ and anti-ChAT for eGFP^+^ fluorochromes comprised about 55% of all the cells, while eGFP^+^ only (green) cells included 30% of all the counted markers and, finally, markers that represented cells labeled by anti-ChAT^+^ (red) only comprised about 15% of all the markers (Fig. 7). A qualitative summary of all the findings has been included in Table 1.

**Table 1:** Shown is a qualitative summary of brain regions expressing ISH ChAT mRNA, along with either ChAT-eGFP or ChAT immunostaining

Region of the brain	Corresponding figures	ChAT-eGFP	Gt-a-ChAT
Forebrain			
Olfactory bulb (Olf)			
Anterior olfactory area (AO)	Figure 1 A, B	+	+
Medial olfactory area (MO)	Figure 1 A, B	+	+
Posterior olfactory area (PO)	Figure 1 A, B	+	+
Endopiriform claustrum (En)	Figure 1 A, B	+	+
Neocortex/Isocortex			
Orbital (O)	Figure 1 A, B	+ +	+
Prelimbic (PrL),	Figures 1 A, B, 3 A–C	+ +	+
Infralimbic (Il),	Figure 1 A, B	+ +	+
Cingulate (Cg),	Figure 1 A, B	+ +	+
Motor (M),	Figure 1 A, B	+ +	+
Somatosensory (S),	Figure 1 A, B	+ +	+
Insular (I)	Figure 1 A, B	+ +	+
Dense cell layer piriform (Pir)	Figure 1 A, B	+ +	+
Dorsal tenia tecta (DTT)	Figure 1 A, B	+ +	–
Basal ganglia and septum			
Medial septum (MS)	Figures 1C, 3 D, F	+ + + +	+ + + +
Horizontal limb of the diagonal band (HDB)	Figure 1 C, D	+ + + +	+ + + +
Ventral limb of the diagonal band (VDB)	Figure 1 C, D	+ + + +	+ + + +
Lateral limb of the diagonal band (LDB)	Figure 1 C, D	+ + + +	+ + + +
Ventral pallidum (VP)	Figure 1 C, D	+ + + +	+ + + +
Caudate putamen (CPu)	Figure 1 C, D	+ + +	+ + +
Globus pallidus (GP)	Figure 1 C, D	+ + +	+ + +
Extension of the amygdala (EA)	Figure 1 C, D	+ + +	+ + +
Tuberculum (Tu)	Figure 1 C, D	+ + +	+ + +
Accumbens nucleus shell (ACB)	Figure 1 C, D	+ + +	+ + +
Hippocampus			
Cornu ammonis (CA)	Figures 1 E, F, 2 A, 4 A, F	+ +	–
Dentate gyrus (DG)	Figures 1 E, F, 2 A, 4 G, I	+ +	–
Amygdaloid regions			
Medial amygdala (Me)	Figures 1 E, F, 2 A	+ +	–
Anterior medial amygdala (MeA)	Figures 1 E, F, 2 A	+ +	–
Posterodorsal medial amygdala (MePD)	Figures 1 E, F, 2 A	+ +	–
Posteroventral medial amygdala (MePV)	Figures 1 E, F, 2 A	+ +	–
Basomedial amygdala nucleus (BMA)	Figures 1 E, F, 2 A	+ +	–
Diencephalon			
Medial habenula (MHb)	Figure 1 F	+ + +	+ + +
Lateral habenula (LHb)	Figure 1 F	+ + +	–
Parafascicular thalamic nucleus (Pf)	Figures 1 E, F, 2 A, B	+ +	+ +
Posterior thalamic nucleus (Po)	Figures 1 E, F, 2 A, B	+ +	+ +
Pretectal thalamic nucleus (PoT)	Figures 1 E, F, 2 A, B	+ +	+ +
Posteromedial thalamic nucleus (PoM)	Figures 1 E, F, 2 A, B	+ +	+ +
Zone incerta (ZI))	Figures 1 E, F, 2 A, B	+ +	+ +
Lateral anterior hypothalamus (LAH)	Figures 1 E, F, 2 A, B	+ +	+ +
Ventrolateral hypothalamus (VLH)	Figures 1 E, F, 2 A, B	+ +	+ +
Anterior hypothalamic nucleus (AHC)	Figures 1 E, F, 2 A, B	+ +	+ +
Lateral hypothalamus (LH)	Figures 1 E, F, 2 A, B	+ +	–
Posterior lateral hypothalamus (PLH)	Figures 1 E, F, 2 A, B; 5 G, I	+ +	+ +
Arcuate nucleus (Arc)	Figure 1 E, F; 2 A, B	–	+ +
Midbrain and Hindbrain			
Pedunculotegmental areas (PTg)	Figure 2 C	+ + +	
Parabigeminal nucleus (PBG)	Figures 2 C, 5 D, F	+ + +	+
Oculomotor nucleus (3 N)	Figure 2 D, E	+ + +	+ +
The motor trigeminal nucleus (5 N)	Figures 2 D, E, 6 D, F	+ + +	+ +
The facial nucleus (7 N)	Figure 2 D, E	+ + +	+ +
The abducens nucleus (6 N)	Figure 2 D, E	+ + +	+ +
The dorsal motor nucleus of the vagus (10 N)	Figure 2 D, E	+ + +	+ +
The hypoglossal nucleus (12 N)	Figure 2 D, E	+ + +	+ +
Inferior colliculus (CIC)	Figures 2 D, 5 A, C	+ + +	–
Dorsomedial tegmental nucleus (DMT)	Figures 2 D; 6 A, C	+ +	–
Laterodorsal tegmental nucleus (LDT)	Figures 2 D, 6 A, C	+	+ +
Ventral tegmental nucleus (VTg)	Figure 2 D	+	+ +
Mesencephalic trigeminal nucleus (Me5)	Figures 2 D, 6 G, I	+	+ +

Only areas demonstrating ISH ChAT mRNA expression are listed

Expression of ChAT-eGFP and Gt-a-ChAT labeling were estimated qualitatively based on both the number of labeled cells and signal strength: +  +  +  + , highest density; +  +  + , high density; +  + , moderate density; + , low density; -, background density

## Discussion

Immunohistochemistry is an important and widely used application to identify cholinergic neurons. The enzyme ChAT acetylates choline to ACh and has been successfully used as a marker to specifically stain cholinergic neurons in several immunohistochemical studies [22, 23]. Nevertheless, this approach seems to have some limitations, and to address these inconsistencies the transgenic mice were developed that express eGFP directed by the promoter for ChAT. The ChAT^BAC^- eGFP experimental mouse model facilitates the identification and study of the cholinergic nerves in the central and peripheral nervous systems [20]. However, we identified discrepancies between ChAT-eGFP expression and immunostaining in several different important brain regions such as the forebrain, diencephalon, and the midbrain when compared to the immunohistochemical presentation of the ChAT antibody.

Our findings of a scattered distribution of ChAT^+^ neurons throughout the entire cortex, with ChAT-eGFP positive neurons outnumbering antibody labeling and scarce overlap of the two markers, is consistent with previous reports [24–26] showing that, in the cortex, co-localization and labeling are lower than in other brain regions [27]. The presence of putative cholinergic neurons in the rodent cortex was first identified through immunolabeling for ChAT [24]. Even those early studies emphasized the limitations in staining sensitivity of these ChAT-immunoreactivity containing neurons throughout the entire cortex, compared to cholinergic neurons in the BF. Thus, this confirmed the reliability of transgenic rodent models with intrinsic eGFP expression specific to ChAT in giving reproducible analysis results for the physiology and function of cortical cholinergic neurons. This was especially true for electrophysiological/cell isolation/tissue-culture studies of cortical cells and circuits and for crucial imaging [28]. Additionally, we observed the strong immunoreactivity of a dense network of fibers in the cortex, which could be masking the weakly labeled ChAT-immunoreactive cell bodies. We observed cortical eGFP-positive neurons clustering mainly into two groups (bipolar and multipolar cells as previously reported [27]) with bipolar appearance constituting most eGFP-positive interneurons. Furthermore, recent studies have emphasized the importance of genetically labeled cholinergic neurons in rodents for studying different subpopulations of the cortical interneurons and providing quantitative information on neuronal distribution and detail patterns of axonal projections [29–31].

The most striking colocalization of ChAT-eGFP expression and anti-ChAT immunoreactivity was seen in the BF cholinergic neurons. These results were consistent when compared to ISH maps from the Allan Brain Institute for Brain Science. Moreover, the BF is composed of various structures that include the extended amygdala (EA) and peripallidal regions, which facilitate processes of cortical activation motivation, attention, learning, and memory [23, 32, 33]. The BF is largely populated by cholinergic neurons compared to other neuronal cell types in rodents and primates, and these neurons project to the cerebral cortex, hippocampus, and amygdala [30, 34]. We found virtually all eGFP-positive neurons were also ChAT-immunoreactive in the entire BF expansion including the MS, VDB/HDB/LDB, VP, and EA. Hence, this confirmed that the transgene recapitulated faithfully the expansion of the endogenous gene, predominantly in areas where ChAT levels were high and also included the CPu, GP, and IPAC. Our findings were also consistent with previous reports that used ChAT-eGFP [27] and ChAT-tauGFP/ChAT-YFP [35] transgenic mice.

According to our observations, eGFP expression could be observed in all areas known to contain cholinergic neurons. In the initial examination of the hippocampus of the ChAT-eGFP mice, dense networks of GFP-containing fibers were found innervating all hippocampal layers, presumably arising mainly from MS-DBB cholinergic projection neurons of the BF [36]. In contrast, these networks were not as dense and significant with antibody labeling. Moreover, intrinsic hippocampal cholinergic interneurons were found almost 30 years ago that may contribute to an intrinsic pool of ACh [37, 38]. Consistent with these findings, we observed a sparse and scattered distribution of eGFP-expressing cholinergic interneurons in the CA1, CA3, and DG subregions. Interestingly, these neurons could not be observed when stained with goat-anti-ChAT antibody, most likely due to the low signal-to-noise ratio of ChAT antibody labeling that showed very weak ChAT immunoreactivity in the hippocampus compared to that in the BF [37]. More recent findings also confirm these observations, reiterating that eGFP [27, 39, 40], or eYFP [35] expression under the control of the *ChAT* promotor is likely to amplify the detection sensitivity of ChAT-expressing cells beyond the detection sensitivity of anti-ChAT antibody. This further confirms that the ChAT^BAC^-eGFP transgenic mouse model could be beneficial for investigating the physiological and functional properties of hippocampal cholinergic interneurons. However, it is yet to be confirmed whether these cholinergic interneuron populations are due to faithful expression of the reporter protein or inappropriate activity of the *ChAT*-BAC promoter constitute [35, 41].

The inferior colliculus (CIC), a midbrain hub for both ascending and descending auditory pathways, contains nicotinic and muscarinic cholinergic receptors throughout, and ACh modulates the responses to acoustic stimuli of a majority of CIC cells [42]. The CIC receives cholinergic inputs mainly from the pedunculopontine tegmental nucleus (PPT) [43]. Nevertheless, reports of intrinsic cholinergic neuronal cell population within the CIC are almost non-existent. We report a significant population of ChAT-eGFP expressing neurons in the CIC but these are not labeled by the antibody.

In 1986 the presence of smaller cholinergic populations (Ch8) was first discovered in the parabigeminal nucleus (PBG) located at the lateral edge of the midbrain [44]. It was also reported that, in the mouse, approximately 80–90% of the PBG cells were cholinergic, which provided a major source of extrinsic cholinergic projections to the superior colliculus [44, 45]. Similarly, in the current study, we observed both ChAT-eGFP expression and antibody labelling in the PBG. However, as we see an overlap of both eGFP and antibody labeling was seen, there were also some cells labeled only with the antibody toward the ventrolateral end of the cholinergic population. PBG contains neurons that lack vesicular ChAT [46] and this might be the reason for some ChAT neurons not expressing eGFP.

Smaller cholinergic populations are also located in the hypothalamus [47, 48]. A substantial proportion of hypothalamic cells were found in the lateral preoptic-hypothalamic continuum the peduncular part of the lateral hypothalamus (LPO-PLH) [49]. In agreement with previous findings, we also report small population of sparingly distributed cholinergic neurons in the PLH. Here we found both ChAT-eGFP expressing and ChAT-immunoreactive cells, with some cells overlapping and some cells expressing either only eGFP or Gt-anti-ChAT-labelling. Again, the differences are most likely accounted for by regional differences and expression fidelity.

The habenula is a complex nucleus, which is a part of the epithalamus that connects the limbic forebrain and the midbrain, and is divided into medial (MHb) and lateral (LHb) subregions [50]. The MHb has drawn attention recently for its dense population of cholinergic neurons and the expression of unique nicotinic ACh receptors (β4 and α5 subunits); thus it is thought to regulate nicotine aversion and withdrawal [51, 52]. In the current study, we observed doubled-labeled neurons with eGFP expression and antibody labeling in the MHb, but lacking of antibody labeling in the LHb. However, the LHb has received considerable attention for its potential role in cognition and pathogenesis of various psychiatric disorders, and since we observed eGFP expression in the LHb, the ChAT-eGFP mouse model could be a potential transgenic model to study further on the habenula complex.

Cholinergic neurons of the midbrain nuclei, such as pedunculopontine nucleus (PPN) and laterodorsal tegmental nucleus (LDT), provide widespread innervation to the thalamus and the basal ganglia [53, 54]. As such, they have been associated with locomotion, reward, arousal, and control of the sleep/wake cycle [55, 56]. In the current study, in agreement with previous findings [53, 54, 57, 58], we report ChAT-immunoreactivity in the LDT region of the midbrain with faint and sparse distribution of eGFP expression. Nevertheless, for the first time, we found strong but small ChAT-eGFP expressing neurons in the dorsomedial tegmental (DMT) area but interestingly no anti-ChAT immunoreactivity (Fig. 6 B), which accounts for the regional specific expression fidelity of eGFP. Hence, the ChAT-eGFP mouse model could be useful for studying the cholinergic system in the DMT.

In our study, we observed that the motor nucleus of the trigeminal-V (5 N) densely packed with cholinergic neurons, as reported recently for the cre-dependent fluorescence reporter mouse line [29]. It is clear from the current study that eGFP expression and antibody labeling overlap for cholinergic neurons in the 5 N for the ChAT-eGFP mouse line, except for some sporadically distributed cholinergic cells with only eGFP expression. The reason for these discrepancies could be the fidelity of the ChAT-eGFP expression compared to goat-anti-ChAT immunoreactivity.

The mesencephalic trigeminal nucleus (Me5), located at the mesopontine junction, contains primary sensory neurons that innervate the muscle spindle of the masticatory muscles in the oro-facial region and is responsible for receiving and transmitting proprioception from this region [59]. There are not many reports about the cholinergic neurons of the Me5. Nevertheless, two reports on rats, state that Me5 contains prominent AChE-reactive cells [60] and ChAT-immunostained neurons [57]. Consistent with these studies, we report both ChAT-eGFP expression and anti-ChAT immunoreactivity in the Me5 region. There was a prominent aggregated neuronal population containing both eGFP and ChAT-immunoreactivity, but we also found a secluded population containing only ChAT-immunoreactivity. Once again, this accounted for regional specific eGFP expression.

## Limitations

While evaluating the expression of eGFP and anti-ChAT-immunoreactivity, we included results from an already published and well-established ISH ChAT mRNA expression database from the Allen Institute (https://alleninstitute.org) which was generated using automated, high-throughput procedures, that is simply not possible or feasible to set up in our laboratories. However, for future large-scale analysis and comparison with other transgenic models, we recommend the use of other techniques, such as RNAscope to confirm and verify the validity and replicability of both antibody specific signals and RNA probe signals.

## Conclusion

In line with these findings, the ChAT^BAC^- eGFP mouse model qualifies as a reliable transgenic model for studying the cholinergic cell population, especially the cortical cholinergic interneurons, stratal cholinergic interneurons, and BF cholinergic projection neurons (Ch1-Ch4). The current study also provides evidence of a significant population of cholinergic interneurons in the hippocampal CA1, CA3 and DG regions visualized through eGFP expression. Our findings further suggest the possible utility of this mouse model to study the mesopontine tegmentum (Ch5 and Ch6), medial habenula nucleus (Ch7), and parabigeminal nucleus (Ch8) cholinergic cell population. In accordance with our findings, the *ChAT*-BAC transgenic mice indicate an excellent tool to determine functional and physiological properties of *bona fide* cholinergic neurons. However, its use to discover novel populations of cholinergic neurons should require caution, due to the ectopic expression of the *ChAT*-BAC promoter, as this mouse line is known to harbor multiple copies of the BAC, and no study up to date has reported information on the integration site. Even though, we found eGFP expression in areas such as the 5 N and Me5, due to discrepancies in fidelity of eGFP expression compared to antibody labeling, using this model for areas such as the brainstem should be reconsidered. This is because; (1) BACs used for the generation of the ChAT-eGFP mouse line might not contain all *cis*-DNA segments essential for appropriate silencing of the cholinergic gene locus in non-cholinergic cells; (2) chromatin elements at the BAC integration site could infrequently override the normal control of the cholinergic gene locus [41]. Moreover, once mRNA copy number for transgene is confirmed and genes of interest encoded by BAC is validated by methods that detect expression of the endogenous genes, quantitative representations of intrinsic *ChAT* and eGFP mRNA co-localization could be feasible. Nevertheless, since the cholinergic system plays an essential role in several pathological conditions, there is no doubt that transgenic mouse models such as the ChAT^BAC^-eGFP model, will aid understanding of the nature of cholinergic transmission in the brain, and mechanistic information regarding the influence of therapeutic interventions on this system.

## Methods

### Animals

The experiments were performed on adult ChAT^BAC^- eGFP transgenic mice (JAX 00790, B6.Cg-Tg(RP23-268L19-EGFP)2Mik/J) JAX of mixed sex purchased from The Jackson Laboratory (JAX), Maine, USA. The mice were bred and housed at Western Sydney University, School of Medicine Animal Facility (Campbelltown, Australia). The creation of the transgenic animals was described elsewhere [20]. The mice were housed in a temperature-controlled environment, with a normal 12:12 h light–dark cycle, with free access to food and water. All mice were monitored daily. All procedures were performed in accordance with the regulations of the Animal Care and Ethics Committee of Western Sydney University (A14717).

Male adult mice (n = 4; two animals for brain mapping, and an additional two animals for imaging purposes) were euthanized with sodium pentobarbitone (0.1 ml, 200 mg/ml) and transcardially perfused with ice-cold saline followed by 4% paraformaldehyde in 0.1 M PBS (100 ml with 4 ml/min rate). Brains were removed and stored in the same fixative at 4◦C for six hours, and then transferred to 30% sucrose in 0.1 M PBS overnight for cryoprotection. Once the brains sank to the bottom of the container, they were embedded and frozen in 4% gelatin in 0.1 M PBS. Using a Leica CM 1950 cryostat, the brains were cut into 40 µm thick coronal sections in three series. One series of sections of the whole brain were collected on gelatin-coated slides, the second and third series were collected in a 0.1 M PBS buffer for further immunohistochemical processing.

We used the second series of sections for immunohistochemical labeling of ChAT. Sections were incubated with normal serum to prevent non-specific binding [3% normal donkey serum (Jackson ImmunoResearch Laboratories, West Grove, PA) in PBS, 2 h]. To visualize ChAT-positive neurons for fluorescent microscopy analysis, the sections were incubated with a goat-anti-ChAT primary antibody (1: 250, Millipore, ab144p, #3429630, for 48 h at 4 ℃) with 0.1% Triton X-100 followed by AF 594 donkey-anti-goat IgG secondary antibody (1:200, Jackson ImmunoResearch Laboratories, #129–709, for two hours at room temperature). The sections were then mounted on gelatin-coated slides and immediately coverslipped with VECTASHIELD antifade mounting medium with DAPI (Vector Laboratories, #H1200).

### Mapping

One of the three series of 40-μm sections through the adult brain was examined. Forty-four coronal sections were mapped by using a Zeiss AxioImager M2 microscope with Apotome (Carl Zeiss Microscopy). Contours were drawn to map the landmark regions of the mouse brain including forebrain, midbrain and hindbrain regions by using the Neurolucida software (MBF Bioscience, VT, USA), guided by the Allen Mouse Brain Atlas [61]. To quantify the number of eGFP^+^, anti-ChAT^+^ and double labelled cholinergic neurons in the transgenic mouse brain, we manually counted the cell bodies using three different markers green (eGFP expression), red (anti-ChAT), and blue (overlap) respectively. The marker totals were averaged between the two mapped brains and presented as percentage fraction of the total markers counted using Prism GraphPad (Version 9) and Microsoft Excel.

### In Situ* hybridization—allen brain institute*

We downloaded and analyzed images of ChAT in situ hybridization (ISH) on adult mouse brain. Images were downloaded from the Allen Institute for Brain Science website [21]and contrast was modified by using Adobe Lightroom 2022 (Adobe). ISH images from the Allen Mouse Brain Atlas showed the ChAT gene expression patterns [21] were aligned in the same two-dimensional coordinate space with the reference atlas created for the project. This allowed a comprehensive comparison of ISH ChAT mRNA expression with either ChAT-eGFP expression or anti-ChAT immunoreactivity [62].

### Confocal imaging

Coverslipped ChAT-eGFP samples stained for ChAT^+^ cholinergic cells were imaged using a Confocal ZEISS laser-scanning microscope (LSM800) with an argon laser and processed using the Zen Blue^®^ software package. The super resolution images were obtained using Airyscan with 1.3 × crop area, and Z-stacks (18 slices; 23.63 µm) were captured using a 20 × (0.8NA) objective and NA1.0 for reflective imaging, (unless otherwise specified). Reflective imaging was achieved using the 561 nm wavelength for ChAT^+^ cells, and 488 nm wavelength for ChAT-eGFP expression. Extended depth of focus images was obtained by collapsing the Z-stacked 3D images of resolution 2146 × 2146 pixels, to better identify the objects and provide greater accuracy.

## Data Availability

The data that support the findings of this study are available from Western Sydney University Research Library, but restrictions apply to the availability of these data, and so they are not publicly available. Data are however available from the corresponding authors upon reasonable request and with the permission of Western Sydney University.

## General text

BAC: Bacterial artificial chromosome
GFP: Green fluorescent protein
eGFP: Enhanced green fluorescent protein
ISH: In situ hybridization
DNA: Deoxyribonucleic acid
ChAT: Choline-acetyltransferase
Ach: Acetylcholine
VAChT: Vesicular acetylcholine transporter
PFA: Paraformaldehyde

## Brain regions

AO: Anterior olfactory area
En: Endopiriform claustrum
O: Orbital cortex
PrL: Prelimbic cortex
Il: Infralimbic cortex
Cg: Cingulate cortex
M: Motor cortex
S: Somatosensory cortex
I: Insular cortex
Pir: Piriform cortex
DTT: Dorsal tenia tecta
OLF: Olfactory areas
BF: Basal forebrain
MS: Medial septum
HDB: Horizontal limb of the diagonal band
VDB: Ventral limb of the diagonal band
LDB: Lateral limb of the diagonal band
(VP): Ventral pallidum
(CPu): Caudate putamen
(GP): Globus pallidus
(EA): Extension of the amygdala
(Tu): Tuberculum
(ACB): Accumbens nucleus shell
CA: Cornu ammonis
DG: Dentate gyrus
Me: Medial amygdala
MeA: Anterior medial amygdala
MePD: Posterodorsal medial amygdala
MePV: Posteroventral medial amygdala
BMA: Basomedial amygdala nucleus
MHb: Medial habenula
LHb: Lateral habenula
Pf: Parafascicular thalamic nucleus
Po: Posterior thalamic nucleus
PoT: Pretectal thalamic nucleus
PoM: Posteromedial thalamic nucleus
ZI: Zone incerta
LAH: Lateral anterior hypothalamus
VLH: Ventrolateral hypothalamus
AHC: Anterior hypothalamic nucleus
LH: Lateral hypothalamus
PLH: Posterior lateral hypothalamus
Arc: Arcuate nucleus
PTg: Pedunculotegmental areas
PBG: Parabigeminal nucleus
PPT: Pedunculopontine tegmental nucleus
PPN: Pedunculopontine nucleus
3 N: Oculomotor nucleus
5 N: The motor trigeminal nucleus
7 N: The facial nucleus
6 N: The abducens nucleus
10 N: The dorsal motor nucleus of the vagus
12 N: The hypoglossal nucleus
CIC: Inferior colliculus
LDT: Laterodorsal tegmental nucleus
DMT: Dorsomedial tegmental nucleus
VTg: Ventral tegmental nucleus
Me5: Mesencephalic trigeminal nucleus
IPAC: Posterior limb of the anterior commissure
LPO-PLH: Lateral preoptic-hypothalamic continuum—peduncular part of the lateral hypothalamus
